# Incidental findings of typical iNPH imaging signs in asymptomatic subjects with subclinical cognitive decline

**DOI:** 10.1186/s12987-021-00268-x

**Published:** 2021-08-14

**Authors:** Doortje C. Engel, Lukas Pirpamer, Edith Hofer, Reinhold Schmidt, Cornelia Brendle

**Affiliations:** 1 Department of Neurosurgery, University Hospital of Tuebingen, Hoppe-Seyler-Strasse 3, 72076 Tuebingen, Germany; 2 Institute for diagnostic and interventional neuroradiology, University Hospital Wuerzburg, Josef-Schneider-Strasse 11, 97080 Wuerzburg,, Germany; 3 Clinical Division of Neurogeriatrics, Department of Neurology, Medical University of Graz, Auenbruggerplatz 22, 8036 Graz, Austria; 4 Department of Neuroradiology, University Hospital of Tuebingen, Hoppe-Seyler-Strasse 3, 72076 Tuebingen, Germany; 5 Institute for Medical Informatics, Statistics and Documentation, Medical University of Graz, Auenbruggerplatz 2, 8036 Graz, Austria

**Keywords:** Idiopathic normal pressure hydrocephalus, Neuropsychological testing, Trail Making Test-B, Tight high convexity, Evans’ index, Short physical performance balance test, Stand score

## Abstract

**Background:**

The etiology of idiopathic normal pressure hydrocephalus (iNPH) remains unclear. Little is known about the pre-symptomatic stage. This study aimed to investigate the association of neuropsychological data with iNPH-characteristic imaging changes compared to normal imaging and unspecific atrophy in a healthy population.

**Methods:**

We extracted data from the community-dwelling Austrian Stroke Prevention Family Study (ASPS-Fam) database (2006–2010). All subjects underwent a baseline and identical follow-up examination after 3–5 years with MR imaging and an extensive neuropsychological test battery (Trail Making Test B, short physical performance balance, walking speed, memory, visuo-practical skills, composite scores of executive function and g-factor). We categorized the subjects into “iNPH”-associated, non-specific “atrophy,” and “normal” based on the rating of different radiological cerebrospinal fluid (CSF) space parameters. We noted how the categories developed over time. We assessed the association of the image categories with the neuropsychological data, different demographic, and lifestyle parameters (age, sex, education, alcohol intake, arterial hypertension, hypercholesterolemia), and the extent of white matter hyperintensities. We investigated whether neuropsychological data associated with the image categories were independent from other parameters as confounders.

**Results:**

One hundred and thirteen subjects, aged 50–70 years, were examined. The imaging category “iNPH” was only present at follow-up. A third of subjects with “atrophy” at baseline changed to the category “iNPH” at follow-up. More white matter hyperintensities (WMH) were present in later “iNPH” subjects. Subjects with “iNPH” performed worse than “normal” subjects on executive function (*p* = 0.0118), memory (*p* = 0.0109), and Trail Making Test B (TMT-B. *p* < 0.0001). Education, alcohol intake, diabetes, arterial hypertension, and hypercholesterolemia had no effect. Age, number of females, and the extent of white matter hyperintensities were higher in “iNPH” than in “normal” subjects but did not significantly confound the neuropsychological results.

**Conclusions:**

Apparent asymptomatic subjects with “iNPH” imaging characteristics presented with subclinical cognitive decline and showed worse executive function, memory, and TMT-B results than “normal” subjects. WMH seem to play a role in the etiology before ventriculomegaly. Clinical screening of individuals with incidental iNPH-characteristic imaging and conspicuous results sof these neurocognitive tests needs further validation.

## Introduction

Idiopathic normal pressure hydrocephalus (iNPH) decreases the health-related quality of life [1]. Treatment by cerebrospinal fluid (CSF) diversion can improve the characteristic symptomatic triad of dementia, gait disturbance, and urinary incontinence and the health-related quality of life [2]. However, patients do not reach the same level as population-matched controls [3]. Other treatment strategies have not been proven effective [4, 5]. Several domains have been investigated without sufficient clarification of the etiology and pathophysiology of iNPH. Specific familial and genetic factors [6–10] and vascular changes such as decreased cerebral blood flow in the periventricular regions [11, 12] are associated with iNPH. Also, the CSF of iNPH patients contains an increased concentration of neurofilament light chain protein together and decreased amyloid precursor protein-derived proteins [13–15]. However, how these factors and parameters interact and cause the clinical development of iNPH remains unknown. According to previous reports, radiological changes can precede apparent symptoms in patients with iNPH [16–19]. Interestingly, a small group of asymptomatic subjects with iNPH-typical imaging findings in one study showed subclinical declines in verbal fluency and motor regulation, whereas mini-mental state examination (MMSE) and Trail Making Test B (TMT-B) were in the normal range [16]. On the other hand, correlating MMSE, semantic fluency tests, and motor programming to radiological data in the same population-dwelling long-term cohort study did show worse scores in “possible” iNPH subjects [17].

From another point of view, the number of diagnostic imaging procedures in clinical routine has increased rapidly in the past decades, leading to an increase of incidental findings [20]. In this view, imaging findings representing brain volume loss or—less often—iNPH-related changes are common; however, asymptomatic subjects often do not receive further investigation or follow-up.

In order to further understand whether imaging signs in asymptomatic patients which are potentially associated with iNPH, coincide with subclinical cognitive alterations and how this develops over time, we analysed prospectively-collected imaging and neurocognitive data from the Austrian Stroke Prevention Family Study (ASPS-Fam; [21–24].

## Methods

### Subjects

Data were extracted retrospectively from the prospectively collected database of the ASPS-Fam. ASPS-Fam represents an extension of the Austrian Stroke Prevention Study (ASPS), which was established in 1991 [24–26]. Study participants of the ASPS and their first-grade relatives were invited to enter ASPS-Fam. Inclusion criteria were no history of previous stroke or dementia and a normal neurologic examination. Summarized, the community-dwelling cohort of the ASPS-Fam consisted of randomly selected individuals aged 50–75 years without neuropsychiatric disease. In total, 400 subjects were enrolled between 2006 and 2010, of whom 385 underwent an MRI. After 3–5 years, the subjects were invited for a follow-up examination. Subjects who had suffered from a stroke in the meantime or reported acute neurologic symptoms were excluded. Concordantly clinical and imaging data of both baseline and follow-up examinations were available in 117 subjects. An additional four subjects were excluded because imaging data revealed post-ischemic defects, a minor intraparenchymal bleeding, a resection defect of unknown origin and a parenchymal defect of unknown origin, and these signs of neurologic diseases might bias the analysis. Finally, we included 113 subjects, each with baseline and follow-up examinations, in the analysis. Not all test parameters were available for each patient, resulting in smaller patient numbers in the respective groups.

### Demographic characteristics and lifestyle parameters

We extracted age, sex, and several treatable clinical factors from the database. Treatable clinical factors included the presence of diabetes (definition: history or current treatment of diabetes or fasting blood glucose level at the examination > 126 mg/dl), HbA1c (mg/dl), amount of alcohol consumption (definition: units of beer, wine or liquor (count double) per day), presence of hypertension (definition: history or current treatment of hypertension or blood pressure readings at the examination > 140/90 mmHg), and hypercholesterolemia (definition: history or current treatment of hypercholesterolemia, total cholesterol at examination > 200 mg/dl or low-density lipoprotein at examination > 130 mg/dl). The education was recorded by a score representing the employment based on the level of education: score 1 = homemaker, farmer, score 2 = clerk, office employee, score 3 = public official; score 4 = academic career (master or more).

### Neurocognitive and motor testing

Subjects underwent testing at baseline and at follow-up 3–5 years later as described previously [24]. In short, Trail Making Test B was conducted for assessing attention and motor speed, and we noted the time needed to complete the test (in seconds). Memory was tested by Bäumler’s Lern- und Gedächtnis Test (LGT-3 [24]) and the visuo-practical skills by Purdue’s Pegboard test [27]. Both parameters were transferred into z-scores; z-score = 0 represents the mean of the study cohort with a standard deviation of 1, meaning that a negative value expresses a worse performance compared to the norm. Additionally, composite z-scores were built to attenuate outliers in individual tests [22]: executive function comprised TMT-B, digit span forward and backward, and Wisconsin Card Sorting Test [28], and intelligence or general fluid cognitive ability factor (g-factor) comprised an extensive neuropsychological test battery [29]. The short physical performance balance test (SPPB) was also conducted [30]. The stand test (max. 4 points) and walking speed test (in m/s) were assessed separately.

### MRI data

MRI examinations were conducted on a 3 T scanner MAGNETOM Trio, A Tim System, (Siemens, Erlangen, Germany) using identical protocols. The examinations consisted of a T2-weighted spin-echo and a T2-weighted fluid attenuation inversion recovery (FLAIR) sequence (RESOLUTION), both performed in the axial direction (resolution of 0.85 × 0.85×3 mm) and a 1 mm isotropic T1-weighted 3D sequence with magnetization prepared rapid gradient echo (MPRAGE). Artifacts with moderate impairment of the image quality were visible in 9 imaging datasets; all images could be assessed.

**Fig. 1:**
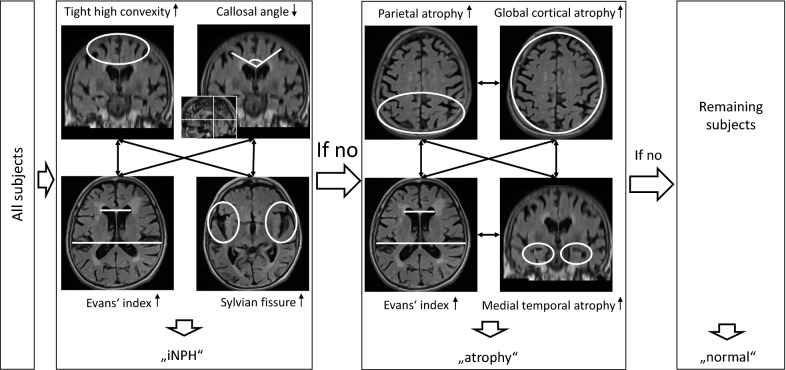
Flow of allocation of the study subjects to the imaging categories. The classification into “iNPH,” “atrophy,” and “normal” based on different combinations of conspicuous liquor space parameter ratings. For the categories “iNPH” and “atrophy,” at least one parameter pair linked by arrows had to be rated as pathological. The callosal angle was measured in a coronal slice perpendicular to the anterior commissure-posterior commissure line [33]

**Fig. 2:**
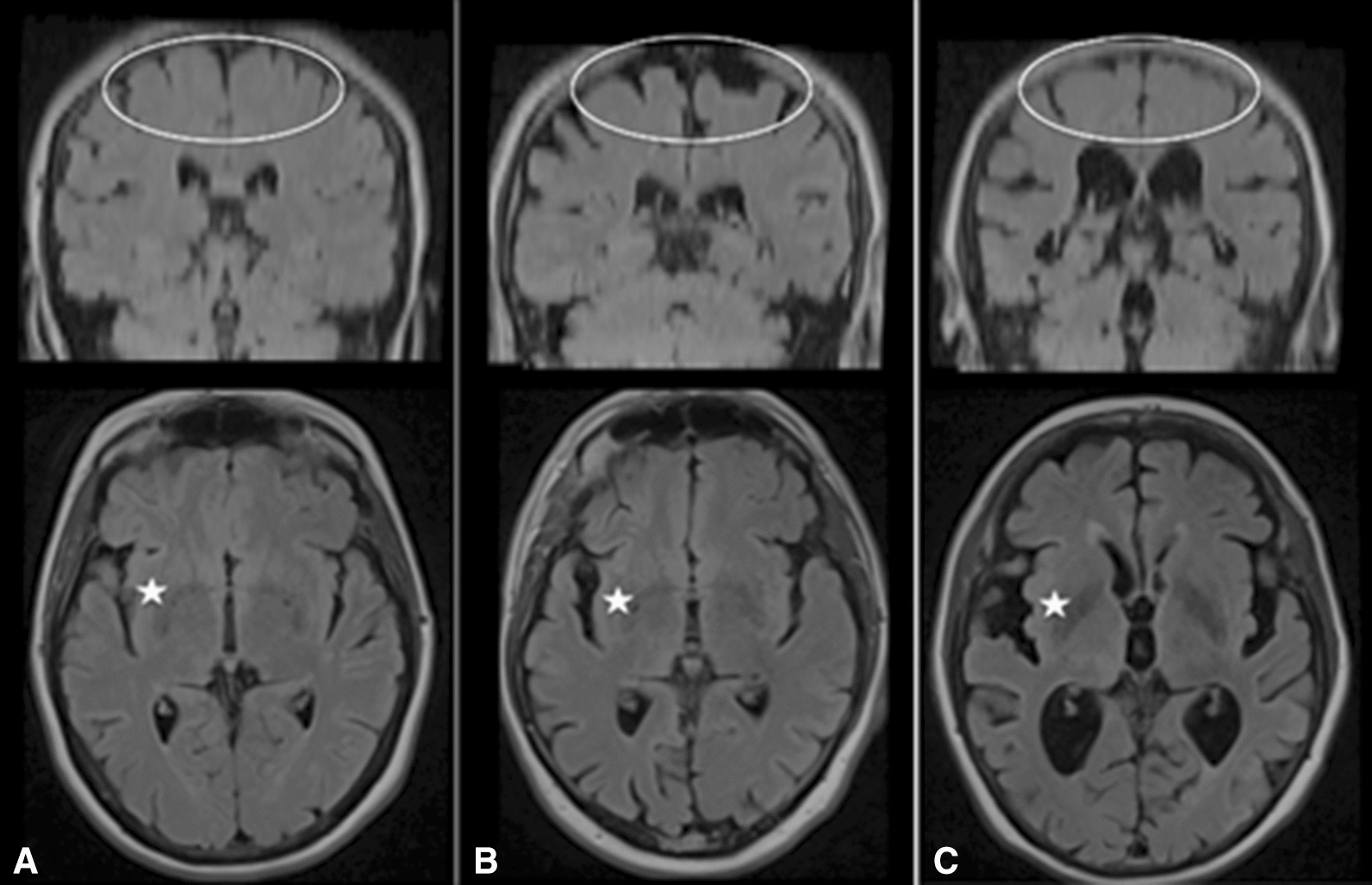
Examples of “normal,” unspecific “atrophy,” and “iNPH” subjects. T2-weighted Fluid inversion recovery (FLAIR) coronal (upper row) and axial (lower row) images of **A** “normal,” **B** unspecific “atrophy” (note atrophy of both the apex, Sylvian fissures, and mild enlargement of the lateral ventricles) and **C** “iNPH” (note the discrepancy between the relatively tight apex, wide Sylvian fissures and ventricles). *Ellipse* shows apex. One of the Sylvian fissures is located left of the *star*

Imaging datasets were displayed in the software Mango 4.0.1 (1510; Lancaster, Martinez, Research Imaging Institute, University of Texas Health Science Center), enabling 3D visualization of the images. We rated the following CSF space parameters at baseline and follow-up with the below-mentioned references as guidelines and templates: Evans’ index in the axial plane (pathological at > 0.3, [31]), tight high convexity (THC) in several contiguous axial and coronal slices (score 0–3 [dilated—severely tight], pathological at score ≥ 2, derived from Fig. 1 in [32]), callosal angle at the posterior commissure in a coronal slice perpendicular to the anterior commissure—posterior commissure plane (pathological at < 90°, [33]), the width of both Sylvian fissures in axial and coronal planes (score 0–3 [narrowed—severely dilated], pathological at score ≥ 2, derived from Fig. 1 in [32]), medial temporal lobe atrophy in the coronal plane (score 0–4 [normal—severe hippocampal atrophy and widened choroid fissure], pathological at score ≥ 2, [34]; see also Fig. 1 in [35]), parietal atrophy in several axial and coronal slices (score 0–3 [normal—knife blade atrophy], pathological at score ≥ 2, derived from Fig. 1 in [36]), and global cortical atrophy in several axial slices (score 0–3 [normal—knife blade atrophy/severely enlarged ventricles], pathological at score ≥ 2, [37]; see also Fig. 1 in [38]). Subsequently, we organized the subjects into three categories according to their imaging findings: “iNPH” (pathological THC or callosal angle (CA), and pathological Evans’ index (EI) or Sylvian fissure (SF), “atrophy” (normal THC and callosal angle, and at least two of the following parameters pathological: Evans’ index, medial temporal lobe atrophy (MTA), parietal atrophy (PA), global cortical atrophy (GCA) and “normal” (all other subjects). We rated subjects with a single pathological parameter as “normal” to consider individual variabilities. Figure 1 shows potential parameter combinations of the three imaging categories and Fig. 2 examples of typical “normal,” “iNPH” and “atrophy” images. We assessed, which subjects at baseline developed characteristics of “iNPH”. As the extent of white matter hyperintensities (WMH) influences cognitive function [39], we assessed the Fazekas score as a potential confounding factor. CB (10 years of experience in diagnostic radiology) assessed the MR images blinded to remaining data. A second reader (NS, six years of experience in diagnostic radiology) rated 20% (n = 48) of the examinations. The interrater reliability of the parameter ratings (normal versus pathological) was Cohens κ = 0.85 for WMH, Cohens κ = 0.88 for Evans’ index, Cohens κ = 0.90 for global cortical atrophy, Cohens κ = 0.92 for medial temporal atrophy, and Cohens κ = 1 for callosal angle, THC, the width of the Sylvian fissure, and parietal atrophy.

### Statistical analysis

Statistical analyses were performed separately for the baseline and follow-up examinations. We calculated the association of age, education, alcohol consumption, WMH, and cognitive test results with the imaging categories by Wilcoxon test at baseline (categories subsequent “iNPH” yes or no) or Kruskal–Wallis test with post-hoc Dunn’s test at follow-up (categories “iNPH,” “atrophy,” “normal”). We tested the association between sex, diabetes, hypertension, and hypercholesterolemia with the imaging findings by two-tailed Fisher’s exact (two categories) test or Chi-Squared test (three categories). We performed multiple logistic regression analyses to identify the independence of associations between the imaging categories and significant neurocognitive test variables from significant confounding parameters. The significance level was set at a *p*-value < 0.05. JMP 13.0 (SAS, Cary, USA) served as statistical software.

## Results

### Imaging categories

At the baseline examination, 93 subjects had “normal” imaging findings, 20 were categorized as “atrophy,” and no subject had “iNPH”-associated imaging characteristics. At the follow-up examination, the category “normal” contained 63 subjects, “atrophy” 42 subjects, and the category “iNPH” 8 subjects.

The category “iNPH” contained two subjects with initially “normal” findings and six subjects with “atrophy” at baseline. A total of 28 initially “normal” subjects developed “atrophy” at follow-up. Table 1 shows the distribution of CSF space parameter ratings in the different categories at follow-up. No subject was categorized as “iNPH” at the baseline examination; thus, we compared the demographic characteristics, lifestyle parameters, and neurocognitive test results of individuals who developed imaging features of “iNPH” at the follow-up examination and the control group without subsequent iNPH-associated findings.

**Table 1:** Tabulation of the number of subjects with conspicuous parameters in the different imaging categories at follow-up

Category	Number	THC	CA	EI	SF	MTA	PA	GCA
iNPH								
	1		x	x	x	x	x	x
	1		x	x		x	x	x
	1		x	x		x		x
	1	x		x	x	x	x	x
	1	x		x		x		
	1	x	x		x	x	x	x
	2	x			x	x		x
Atrophy								
	5			x	x	x	x	x
	1			x	x	x		x
	3			x		x	x	x
	1			x		x	x	
	2			x			x	x
	1			x			x	
	1			x				x
	2				x	x	x	x
	1				x	x	x	
	4				x		x	x
	6					x	x	x
	6					x	x	
	9						x	x
Normal								
	1				x		x	
	1				x			x
	1				x			
	1	x					x	
	1					x		
	21						x	
	37							

*iNPH* category with imaging findings resembling idiopathic normal pressure hydrocephalus, *THC* increased tight high convexity score ≥ 2, *CA* callosal angle < 90°, *EI* Evans’ index > 0.3, *SF* enlarged Sylvian fissures score ≥ 2, *MTA* mesial temporal atrophy score ≥ 2, *PA* parietal atrophy score ≥ 2, *GCA* global atrophy score ≥ 2

### Demographic characteristics and lifestyle parameters of the subjects

Age was significantly higher in the follow-up categories “atrophy” and “iNPH” compared to “normal” (*p* = 0.0002 and *p* = 0.0205, respectively). WMH score was significantly higher in subjects at baseline developing “iNPH” later on (*p* = 0.0255) as well as in the categories “atrophy” and “iNPH” compared to “normal” at follow-up (*p* = 0.0470, and *p* = 0.0027, respectively). The sex distribution differed significantly between the categories “iNPH” compared to “atrophy” and “normal” at follow-up (*p* = 0.0283 and *p* = 0.0494, respectively). Therefore, we considered them as potential confounders in further analyses. Tables 2, 3, and 4 tabulate the details for all parameters at both time points and the comparative tests between the categories.

**Table 2:** Baseline characteristics of subjects with and without subsequent development of “iNPH” imaging changes

Parameter	No “iNPH”	Subsequent “iNPH”	Number of subjects	*p*-value
Total (n)	105	8	113	
Age (years, mean ± SD)	64 ± 10	70 ± 3	113	NS
Sex (female/male, n)	51/54	7/1	113	NS
Education (score, mean ± SD)^a^	2.5 ± 1.0	2.0 ± 1.1	113	NS
Alcohol (daily units, mean ± SD)	1.0 ± 0.9	1.3 ± 1.0	111	NS
Diabetes (y/n, n)	11/93	3/5	112	NS
Hypertension (y/n, n)	66/38	7/1	112	NS
Hypercholesterolaemia (y/n, n)	78/26	7/1	112	NS
WMH (Fazekas score, mean ± SD)	1.6 ± 1.2	2.9 ± 1.7	113	0.0255*
SPPB (score, mean ± SD)^b^	3.9 ± 0.3	3.6 ± 0.7	113	NS
TMT-B (sec, mean ± SD)	103 ± 43	150 ± 60	113	0.0199*
Walking speed (m/s, mean ± SD)	0.4 ± 0.1	0.4 ± 0.1	113	NS
Executive function (z-score, mean ± SD)	0.1 ± 0.6	0.1 ± 0.3	51	NS
g-factor (z-score, mean ± SD)	0.1 ± 1.1	0.1 ± 0.8	51	NS
Memory (z-score, mean ± SD)	0.1 ± 1.1	0.0 ± 0.9	54	NS
Visuopractical Skills (z-score, mean ± SD)	0.0 ± 0.8	0.5 ± 0.8	54	NS

iNPH: idiopathic normal pressure hydrocephalus-associated imaging findings; SD: standard deviation; y/n: present yes or no; WMH: white matter hyperintensities; SPPB: short physical performance balance; TMT-B: Trail Making Test B

^a^range of education score: 1 (homemaker, farmer)—4 (academic career)

^b^range of SPPB score: 1–4

*significant difference

**Table 3:** Characteristics of the subjects in relation to the image category at follow-up

Parameter	“Atrophy”	“Normal”	“iNPH”	Number of subjects
Total (n)	42	63	8	113
Age (years, mean ± SD)	74 ± 7	67 ± 10	77 ± 5	113
Sex (female/male, n)	19/23	32/31	7/1	113
Education (score, mean ± SD)^a^	2.5 ± 1.0	2.5 ± 1.0	2.0 ± 1.1	113
Alcohol (daily units, mean ± SD)	1.1 ± 1.0	0.8 ± 0.8	0.7 ± 0.8	107
Diabetes (y/n, n)	5/36	4/57	1/6	109
Hypertension (y/n, n)	19/22	33/29	4/3	110
Hypercholesterolaemia (y/n, n)	26/15	43/19	6/1	110
WMH (Fazekas score, mean ± SD)	2.5 ± 1.5	1.8 ± 1.1	3.8 ± 1.6	113
SPPB (score, mean ± SD)^b^	3.6 ± 0.7	3.8 ± 0.4	3.3 ± 1.0	99
TMT-B (sec, mean ± SD)	139 ± 75	110 ± 48	194 ± 53	109
Walking speed (m/s, mean ± SD)	0.4 ± 0.1	0.4 ± 0.1	0.4 ± 0.1	108
Executive function (z-score, mean ± SD)	− 0.0 ± 0.7	0.2 ± 0.6	− 0.5 ± 0.5	74
g-factor (z-score, mean ± SD)	− 0.3 ± 1.1	0.3 ± 0.9	− 0.8 ± 0.8	72
Memory (z-score, mean ± SD)	− 0.2 ± 1.1	0.2 ± 0.9	− 1.1 ± 0.7	103
Visuopractical Skills (z-score, mean ± SD)	− 0.3 ± 0.8	0.3 ± 0.8	− 0.6 ± 1.2	107

iNPH: idiopathic normal pressure hydrocephalus-associated imaging findings; SD: standard deviation; WMH: white matter hyperintensities; SPPB: short physical performance balance; TMT-B: Trail Making Test B

^a^range of education score: 1 (homemaker, farmer)—4 (academic career)

^b^range of SPPB score: 1–4

**Table 4:** Differences of the study parameters between the imaging categories at follow-up

Parameter	Overall^a^	“atrophy” vs. “normal”^b^	“atrophy” vs. “iNPH”^b^	“iNPH” vs. “normal”^b^
Age (years)	< 0.0001*	0.0002*	NS	0.0205*
Sex (n)	NS	NS	0.0283*	0.0494*
Education (score)	NS	NS	NS	NS
Alcohol (daily units)	NS	NS	NS	NS
Diabetes (n)	NS	NS	NS	NS
Hypertension (n)	NS	NS	NS	NS
Hypercholesterolaemia (n)	NS	NS	NS	NS
WMH (Fazekas score)	0.0009*	0.0470*	NS	0.0027*
SPPB (score)	NS	NS	NS	NS
TMT-B (sec)	0.0020*	NS	NS	0.0036*
Walking speed (m/s)	NS	NS	NS	NS
Executive function (z-score)	0.0129*	NS	NS	0.0210*
g-factor (z-score)	0.0318*	NS	NS	NS
Memory (z-score)	0.0068*	NS	NS	0.0155*
Visuopractical Skills (z-score)	0.0015*	0.0043*	NS	NS

*iNPH* idiopathic normal pressure hydrocephalus-associated imaging findings, *WMH* white matter hyperintensities, *SPPB* short physical performance balance, *TMT-B* Trail Making Test B

^a^Kruskal–Wallis test in continuous parameters, Fisher’s exact test or Chi-Squared test in the remaining parameters

^b^Dunn’s test in continuous parameters, Chi-Squared test in the remaining parameters

*significant difference

### Association of neurocognitive and motor test parameters with the imaging categories

At baseline, subjects later categorized as “iNPH” needed significantly more time to complete TMT-B than the control group (*p* = 0.0199); Table 2 shows the results of all parameters. At follow-up, subjects of the category “iNPH” needed significantly longer to complete TMT-B (*p* = 0.0036) and reached significantly lower z-scores than “normal” subjects in executive function (*p* = 0.0210) and memory (*p* = 0.0155). The same test results did not differ significantly between “iNPH” and “atrophy” subjects and between “atrophy” and “normal” subjects. On the other hand, the visuo-practical skills z-score differed significantly between “normal” and “atrophy” subjects (*p* = 0.0043), but not between “iNPH” and “normal” or “atrophy” subjects. Tables 3 and 4 tabulate details for all parameters. In multiple regression analyses of parameters differentiating significantly “iNPH” from “normal” subjects—namely, TMT-B, executive function, and memory—each remained as significant factors independent from the confounders age, sex, and WMH (*p* < 0.0001, *p* = NS, and *p* = 0.0109, respectively).

## Discussion

Our current data show that subjects with iNPH-associated imaging signs show subclinical deficits with worse performance on mainly frontal lobe function parameters, such as executive function, memory, and TMT-B, compared to subjects without conspicuous imaging findings. Even before iNPH-associated imaging criteria were fulfilled, these subjects inclined to worse neuropsychological performance. This is in accordance with previous studies reporting that imaging signs can precede clinically apparent symptoms [17, 18, 40, 41]. Also, our future “iNPH” imaging subjects had more WMH than normal subjects, leading to the suspicion that WMH might be part of the etiology of iNPH. WMH were present before the lateral ventricles became wider at follow-up. It is known that pulsatility in the CSF space is increased in iNPH patients [15, 42]. Investigations of the glympathic system in iNPH patients using DTI and MR spectroscopy showed altered function of the glymphatic system, in part shown by an accumulation of macromolecules and a decreased leven of N-acetyl aspartate [43–45]. Our current findings of early presence of WMH can support the abnormal findings of the glympathic system in iNPH patients and seem to point into this direction for the etiology of iNPH.

Next to that, a third of subjects with signs of (cortical) atrophy at baseline developed “iNPH” at follow-up. The underlying principle of dynamic pathophysiological processes over ten years was already shown previously [17, 18]. Our and previous findings emphasize the need for understanding that the development of iNPH-associated imaging is a dynamic process and that one-time imaging should not put a subject in a fixed “atrophy” or “normal” box.

In partial accordance with our results, Iseki and colleagues did not find significant differences in MMSE and TMT-B results, but in semantic fluency tests and motor programming comparing subjects with asymptomatic ventriculomegaly with iNPH-features and age-matched controls [16]. Different categorizations of imaging findings were conducted compared to our criteria, and other specific tests were used to investigate neurocognitive function.

Evaluating the neurocognitive test results, we did not find a specific subclinical deficit defining subjects with iNPH-associated imaging findings, particularly differentiating them from subjects with unspecific atrophy. However, this would have been surprising considering only discrete neurocognitive and imaging changes in a healthy population like ours.

Nonetheless, we found a lower performance of “iNPH” subjects than subjects with normal imaging findings in several cognitive tests. In this respect, the most striking test was TMT-B, which already at baseline tended towards worse results in subjects developing “iNPH” later. Hence, TMT-B testing is not only a valuable diagnostic tool in symptomatic probable iNPH patients [46–48] but might also be helpful to search for subtle preclinical abnormalities. Iseki and colleagues did not reveal conspicuous TMT-B results in presymptomatic iNPH patients. This may be due to a different composition of their study cohort [16]. Therefore, further studies might reliably confirm our finding. Executive function and memory, functions connected with TMT-B, were significantly worse in subjects with iNPH-associated imaging criteria only at the follow-up time point of the study and not in advance. Though, they still might be helpful to identify subjects at risk for developing clinically evident iNPH.

A typical clinical sign of iNPH, the short-stepped, magnetic gait, is present in approximately 90% and cognitive impairment in 50% of iNPH patients [49]. However, a previous study showed that only 50% of symptomatic iNPH subjects reported gait problems as a symptom during their first visit, and only 35% reported cognitive symptoms [50]. There seems to be a discrepancy between test results and subjective opinions of patients. Based on the current data, gait problems do not arise early in the timeline of the etiology of potential iNPH. Therefore, gait and balance testing by walking speed and the SPPB stand score seem not to be an effective early screening tool, though more sensitive gait tests might identify slighter dysfunctions.

Age did not confound the association between neuropsychological test results and the image categories in our study. Though, age has a marked influence on neurocognitive function in general, and the prevalence of iNPH increases with age [50, 51]. Accordingly, none of our subjects presented with iNPH-associated imaging findings at baseline (mean age: 65 ± 10 years), but specific individuals with prior normal imaging or unspecific signs of atrophy developed an iNPH-associated imaging pattern over the study course of five years.

Lifestyle factors that correlated with iNPH in previous literature did not show an association in the current study. Arterial hypertension is the best-documented risk factor associated with symptomatic iNPH according to the International Society for Hydrocephalus and Cerebrospinal Fluid Disorders (ISHCSF) task force and others [15, 52–54]. The lacking association of hypertension with “iNPH” in our study could be due to the small number of affected subjects or maybe only a minor role in the etiology compared to symptomatic iNPH patients. Therefore, as also stated by the ISHCSF task force, it is uncertain whether treatment of arterial hypertension reduces the risk of iNPH or improves the outcome [15]. Kuriyama and colleagues even showed a lower prevalence of hypertension (40%) in their iNPH cohort [50], whereas the Japanese prevalence of hypertension is approximately 60% in the same age group [55].

Other lifestyle-dependent and treatable health factors such as diabetes, hypercholesterolemia, and alcohol intake did not associate with potential iNPH subjects in our study. Omitting lifestyle and treatable comorbidities lead to pathophysiological considerations in the direction of genetic factors [10, 56].

Our results are limited by the fact that the “iNPH” and “atrophy” groups were small, and not all parameters were available in each subject. The subjects did not display the complete pattern of iNPH, but only slight and partial imaging characteristics consistent with their asymptomatic clinical condition. Additionally, particularly chronic neurodegenerative diseases, not visible in diagnostic imaging, were not evident to the researchers and could have biased results. Neuropsychological testing was not corrected for the educational level, which could have influenced performance; however, the educational level did not differ between imaging categories. Next to that, the authors assumed that the imaging signs are strongly correlated with iNPH, due to parallel findings from previous studies from confirmed iNPH patients, without knowing whether these subjects actually would develop symptomatic iNPH. However, the used imaging parameters are associated with iNPH in the literature and no definite non-invasive clinical or imaging criteria to define iNPH exist. Furthermore, the scoring and cut-offs used in this study rely on subjective measurements and empirical experience and might be prone to bias.

## Conclusion

TMT-B performance can already be worse before iNPH-characteristic imaging becomes visible. Apparent asymptomatic subjects with “iNPH” imaging characteristics present subclinical cognitive decline and show worse executive function, memory, and TMT-B results than “normal” subjects. WMH seem to play a role in the etiology of iNPH. Clinical screening of individuals with incidental iNPH-characteristic imaging and conspicuous results of these neurocognitive tests needs further validation. The development of atrophy and iNPH-imaging characteristics is dynamic.

## Data Availability

The datasets used during the current study are available from the corresponding author on reasonable request.

## Abbreviations

ASPS-Fam: Austrian Stroke Prevention Family Study
FLAIR: Fluid attenuation inversion recovery
iNPH: Idiopathic normal pressure hydrocephalus
ISHCSF: International Society for Hydrocephalus and Cerebrospinal Fluid Disorders
LGT-3: Bäumler’s Lern- und Gedächtnis Test (memory test)
MMSE: Mini mental state examination
MPRAGE: Magnetization prepared rapid gradient echo
SPPB: Short physical performance balance test
TMT-B: Trail Making Test B
THC: Tight high convexity
WMH: White matter hyperintensities
